# Formation of Lignin Nanoparticles by Combining Organosolv Pretreatment of Birch Biomass and Homogenization Processes

**DOI:** 10.3390/molecules23071822

**Published:** 2018-07-23

**Authors:** Leonidas Matsakas, Anthi Karnaouri, Andrzej Cwirzen, Ulrika Rova, Paul Christakopoulos

**Affiliations:** 1 Biochemical Process Engineering, Division of Chemical Engineering, Department of Civil, Environmental and Natural Resources Engineering, Luleå University of Technology, 971-87 Luleå, Sweden; leonidas.matsakas@ltu.se (L.M.) anthi.karnaouri@ltu.se (A.K.); ulrika.rova@ltu.se (U.R.); 2 Structural Engineering, Division of Structural and Fire Engineering, Department of Civil, Environmental and Natural Resources Engineering, Luleå University of Technology, 971-87 Luleå, Sweden; andrzej.cwirzen@ltu.se

**Keywords:** lignin nanoparticles, micro-particles, birch, organosolv pretreatment, biomass fractionation, homogenization

## Abstract

Valorization of lignocellulosic biomass into a biorefinery scheme requires the use of all biomass components; in this, the lignin fraction is often underutilized. Conversion of lignin to nanoparticles is an attractive solution. Here, we investigated the effect of different lignin isolation processes and a post-treatment homogenization step on particle formation. Lignin was isolated from birch chips by using two organosolv processes, traditional organosolv (OS) and hybrid organosolv-steam explosion (HOS-SE) at various ethanol contents. For post-treatment, lignin was homogenized at 500 bar using different ethanol:water ratios. Isolation of lignin with OS resulted in unshaped lignin particles, whereas after HOS-SE, lignin micro-particles were formed directly. Addition of an acidic catalyst during HOS-SE had a negative impact on the particle formation, and the optimal ethanol content was 50–60% *v*/*v*. Homogenization had a positive effect as it transformed initially unshaped lignin into spherical nanoparticles and reduced the size of the micro-particles isolated by HOS-SE. Ethanol content during homogenization affected the size of the particles, with the optimal results obtained at 75% *v*/*v*. We demonstrate that organosolv lignin can be used as an excellent starting material for nanoparticle preparation, with a simple method without the need for extensive chemical modification. It was also demonstrated that tuning of the operational parameters results in nanoparticles of smaller size and with better size homogeneity.

## 1. Introduction

Transition to a more sustainable society requires the use of renewable and sustainable resources for the production of fuels, chemicals, and materials. Among the different options, lignocellulosic biomass offers an important alternative as it consists of a renewable and plentiful resource [1]. Lignocellulosic biomass can be derived from a variety of sources, such as residues and by-products from forestry and agricultural sectors. The use of residues and by-products has many advantages, as they do not compete directly with the production of food and feed. Furthermore, their conversion to fuels, chemicals, and materials could become an extra source of revenue for the forestry and agricultural sectors, contributing to the support of rural economies.

Lignocellulosic biomass consists mainly of cellulose, hemicellulose, and lignin and its rigid structure makes it necessary to apply a pretreatment step prior to any further conversion. A number of different pretreatment methods, such as steam explosion, hydrothermal, alkaline, and dilute acid, have been successfully applied to lignocellulosic biomass prior to enzymatic saccharification [2,3,4,5]. Currently, the most common approach for biomass bioconversion involves the fermentation of sugar to ethanol or other fuel after biomass pretreatment and saccharification [6]. Conversion of hemicellulose (consisting mainly of pentoses) to ethanol has been a challenge as *Saccharomyces cerevisiae*, which is commonly used as fermenting organism, lacks the ability to naturally take up pentoses [7]. This has resulted in extended research efforts to construct strains capable of co-utilizing glucose and pentoses aiming to improve the overall ethanol yield [8]. A consequence of the so called ‘glucocentric’ approach is the underutilization of lignin, which is recovered at the end of the process as a low-value by-product and is normally burnt for the production of heat and electricity [9]. Moreover, combustion of lignin raises environmental concerns as it can result in the generation of organic pollutants, oxygenated PAHs, and particulate matter [10]. Lignin, however, can account for up to 40% of the plant dry biomass [6] and techno-economic analysis of lignocellulosic biorefineries have stressed its valorization as an essential step [11].

Lignin is the largest renewable source of aromatics [12] and its complex and diverse nature, rich in functional groups, makes it a very interesting molecule to be used in high added-value applications. These include the production of fuels (e.g., gasoline-range aromatics and alkanes), fine chemicals (e.g., benzene and adipic acid), and materials (e.g., carbon fibers, adsorbents, composites, and polymers), which prompted intense research in this field during the last years [6,12,13,14,15]. Use of the lignin fraction in these processes is a prerequisite for establishing a sustainable biomass biorefinery, whereby all the main biomass components are routed towards product formation. The use of lignin in high-end applications depends on the ability to obtain high-quality lignin fractions. One approach is to recover lignin at the beginning of the process prior to any further conversion of the carbohydrate fractions. This can be achieved by establishing fractionation technologies, in which lignocellulosic biomass is fractionated to relative clean streams of cellulose, hemicellulose, and lignin. These, can be converted to a wide portfolio of products through biological or (thermo)-chemical transformations. Among the different alternatives, organosolv pretreatment offers one of the most promising options for a biomass fractionation process and has already been used for the fractionation of both agricultural residues [1,16,17] and forest biomass [9,16,18]. Organosolv pretreatment utilizes aqueous-organic solvent mixtures for the cooking of biomass at elevated temperatures (100–250 °C) with or without the addition of acid catalyst [19,20,21]. Organosolv pretreatment is advantageous over other lignin isolation processes (e.g., kraft and sulfite process) as it utilizes sulfur-free chemicals (thus not incorporating sulfur into lignin) and retains the majority of the β-ether bonds, resulting in a lignin structure close to natural lignin [12,15,22].

Lignin valorization for the production of chemicals can nevertheless be challenging due to its complex and non-homogeneous structure, which is also affected by the chosen lignin isolation method [23]. Utilization of lignin for the formation of nanostructured materials may overcome this issue [24] as it avoids complex depolymerization and upgrading processes. This approach has already resulted in the preparation of various forms of lignin nanostructures, such as nanolignins, colloidal nanospheres, and nanocapsules [25]. The ensuing lignin micro- and nanoparticles have various applications in material science for improving the mechanical, thermal stability, barrier, antibacterial, and antioxidant properties of polymer nanocomposites, as drug carriers, in the delivery of hydrophobic molecules, and as UV barrier [23,24,26]. Various approaches have been proposed for the formation of lignin particles, including self-assembly, chemical, mechanical, or ultrasonic treatments [27]. Mechanical treatments such as homogenization have already been effectively used for the formation of lignin particles, often in relatively simple and straightforward processes [25,26]. The use of solvents is common [28,29] and important for micro- and nanoparticles formation [25]. Crucially, it overcomes the intrinsic poor solubility of kraft lignin in organic solvents, which requires the application of laborious chemical modifications such as acetylation and grafting to improve yields [25]. Besides adding up to/increasing the process complexity, such modifications often employ environmentally hazardous chemicals [28]. Organosolv lignin has higher solubility in organic solvents, making it a better candidate for these processes.

The aim of the current work was to study the formation of micro- and nanoparticles from organosolv-isolated lignin from birch chips. We assessed the effect of operating conditions of the organosolv fractionation process on the ability to form lignin particles. Specifically, we compared a traditional organosolv fractionation method (OS) that was optimized for birch biomass by our group [18] with a newly developed hybrid organosolv steam-explosion fractionation method (hybrid, HOS-SE) [9]. The effects of the explosive discharge (occurring in the hybrid fractionation process), ethanol content, and addition of acidic catalyst during fractionation on particle formation were examined. Subsequently, we evaluated the effect of homogenization as a post-treatment step on the formation of lignin particles under various ethanol:water ratios and in relation to the choice of lignin isolation process.

## 2. Results and Discussion

### 2.1. Effect of the Fractionation Process on Particle Formation

**Figure 1:**
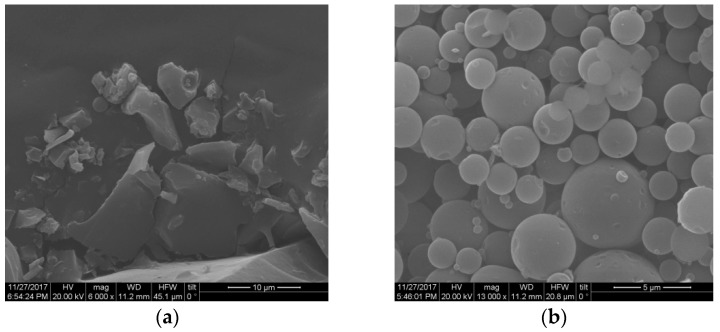
Morphology of lignin after HOS-SE pretreatment, (**a**) with sulfuric acid catalyst; (**b**) without sulfuric acid catalyst. Pretreatment took place at 200 °C for 15 min with 60% *v/v* ethanol content.

Initially, we evaluated the effect of various operational conditions of the HOS-SE fractionation process on the direct formation of lignin particles. Addition of acidic catalysts, such as sulfuric or phosphoric acid, is common during organosolv as it can improve the cellulose content, saccharification, and delignification yields [9,30,31]. Use of acid during pretreatment has also an impact on lignin itself, as it can promote the cleavage of ether bonds [32,33], causing chemical changes to the lignin structure. Here, addition of sulfuric acid (1% *w/w*_biomass_) during organosolv pretreatment promoted the formation of unshaped lignin, whereas absence of the acid catalyst favored the formation of lignin micro-particles (Figure 1). The latter, however, appeared non-homogenous in size, varying mainly from a few μm to >1 μm in diameter, whereas few particles were also smaller than 1 μm. Importantly, this finding demonstrated that spherical lignin particles could be isolated directly from the liquor after pretreatment, without any need for further processing.

**Figure 2:**
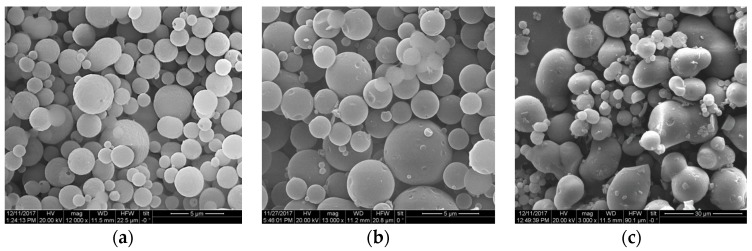
Morphology of lignin after HOS-SE pretreatment with increasing ethanol content of (**a**) 50% *v/v*; (**b**) 60% *v/v*; (**c**) 70% *v/v*.

Lignin is generally insoluble in water [18], but it also contains several hydrophilic functional groups, such as carboxylic and aliphatic hydroxyl groups [34], which give it an amphiphilic nature. Solvent type and content play important roles during the assembly of lignin nanoparticles [28,35]. Therefore, we hypothesized that the ethanol content during pretreatment might affect the formation of lignin nanoparticles. To this end, we evaluated the formation of lignin nanoparticles at an ethanol content varying from 50% to 70% *v/v*. We observed that an ethanol content of 50–60% *v/v* promoted the formation of lignin micro-particles with a well-defined spherical shape (Figure 2a,b). Use of a higher ethanol content resulted in larger particles, but they lacked a defined spherical shape, as indicated by spherical, oval, and undefined appearances (Figure 2c).

**Figure 3:**
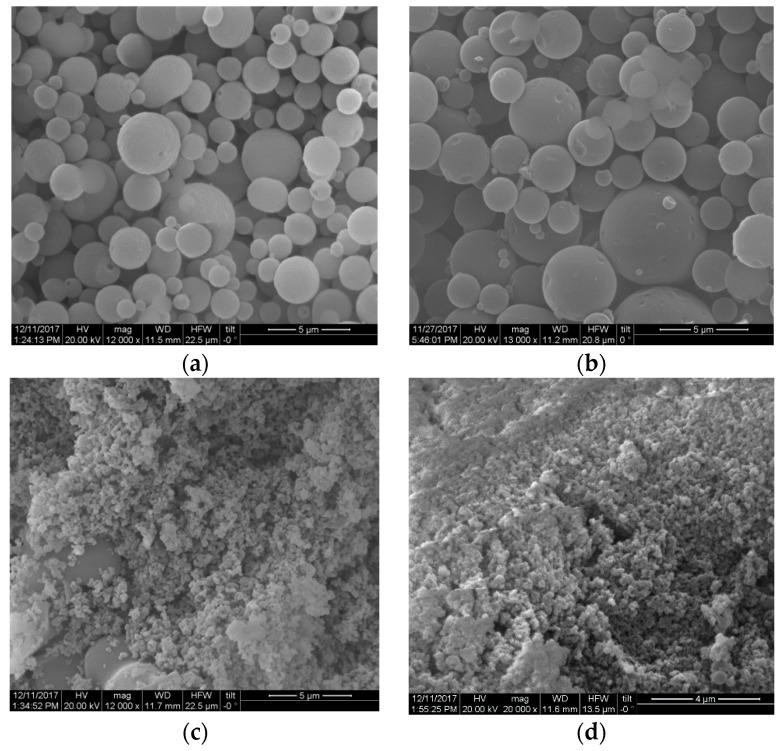
Morphology of lignin after HOS-SE pretreatment with ethanol content of (**a**) 50% *v/v* or (**b**) 60% *v/v*; and after traditional OS pretreatment without explosive discharge with ethanol content of (**c**) 50% *v/v* or (**d**) 60% *v/v*.

Application of high-pressure conditions during homogenization has been used previously for the fabrication of lignin particles [25,26]. For this reason, we suspected that the tension on the solubilized lignin elicited by the explosive discharge step could drive the formation of lignin micro-particles during pretreatment. To test this hypothesis, we compared the morphology of lignin particles with and without the inclusion of the explosive discharge step. For this purpose, we assessed lignin isolated with 50% *v/v* or 60% *v/v* ethanol. Compared to HOS-SE pretreatment (Figure 3a,b), traditional OS (without the explosive discharge step) failed to yield spherical lignin particles, resulting instead in amorphous undefined shapes (Figure 3c,d). The outcome was not affected by the ethanol content. Thus, the forces created during the explosive discharge appear responsible for particle formation during pretreatment. 

### 2.2. Lignin Particle Formation through Homogenization

Homogenization has been used for the preparation of lignin nanoparticles from kraft and organosolv lignin using either a shear homogenizer [26] or a homogenizer [25]. During the process, formation of lignin particles was reported to be significantly affected by ethanol content [25], but not by homogenization time, as long as sufficient time was provided. Accordingly, we investigated the effect of homogenization at different ethanol contents (0% *v/v*, 50% *v/v*, and 75% *v/v*). To ensure that adequate time was given for particles to form, homogenization was performed by recycling the liquid five times.

**Figure 4:**
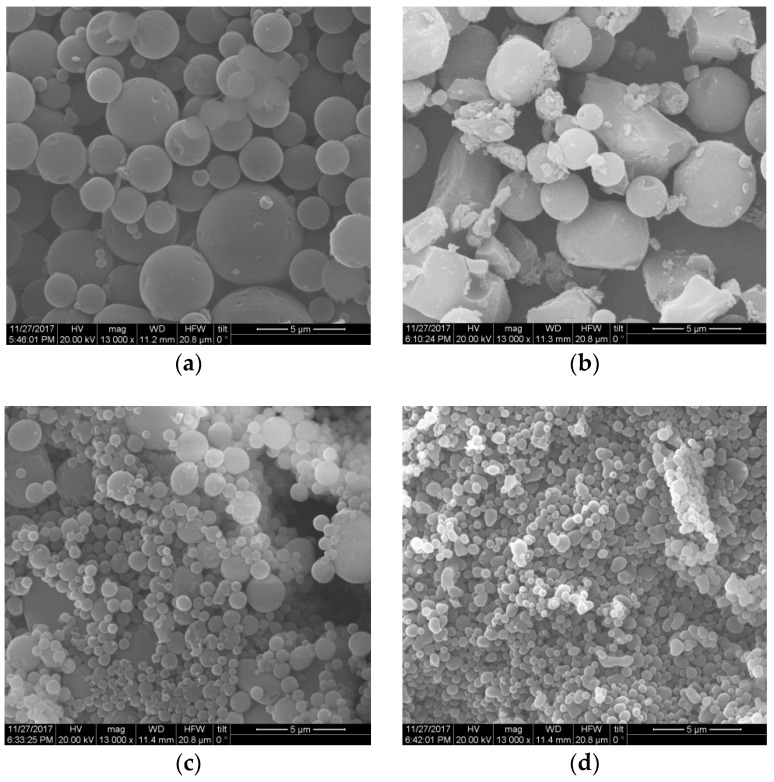
Morphology of lignin isolated with HOS-SE pretreatment with 60% *v/v* ethanol content (**a**) and after homogenization at 500 bar in ethanol/water solution with ethanol content of (**b**) 0% *v/v*; (**c**) 50% *v/v*; and (**d**) 75% *v/v*.

To determine whether post-treatment by homogenization affected the formation of micro- and nanoparticles of different shapes or sizes, we used lignin isolated by both OS and HOS-SE methods. Specifically, we sought to determine the effect of homogenization on amorphous lignin (OS lignin) and on size reduction of already formed particles (HOS-SE lignin). Treatment of HOS-SE lignin (isolated with 60% *v/v* ethanol) by homogenization at 50% *v/v* and 75% *v/v* ethanol content significantly reduced particle size and improved their homogeneity (Figure 4). Specifically, prior to homogenization, HOS-SE lignin (Figure 4a) consisted mainly of micro-particles of 1–3 μm, with a few having slightly higher or lower diameter. After homogenization with 50% *v/v* ethanol, smaller particles with a diameter ranging from 1 μm to approximately 250–300 nm were formed (Figure 4c). Increasing ethanol content during homogenization to 75% *v/v*, further reduced particle size, with the majority of them now ranging between 250 nm and 500 nm (Figure 4d). On the contrary, homogenization without ethanol caused deformation of already spherical particles (Figure 4b). A similar homogenization outcome was observed also with HOS-SE lignin isolated with 50% *v/v* and 70% *v/v* ethanol.

**Figure 5:**
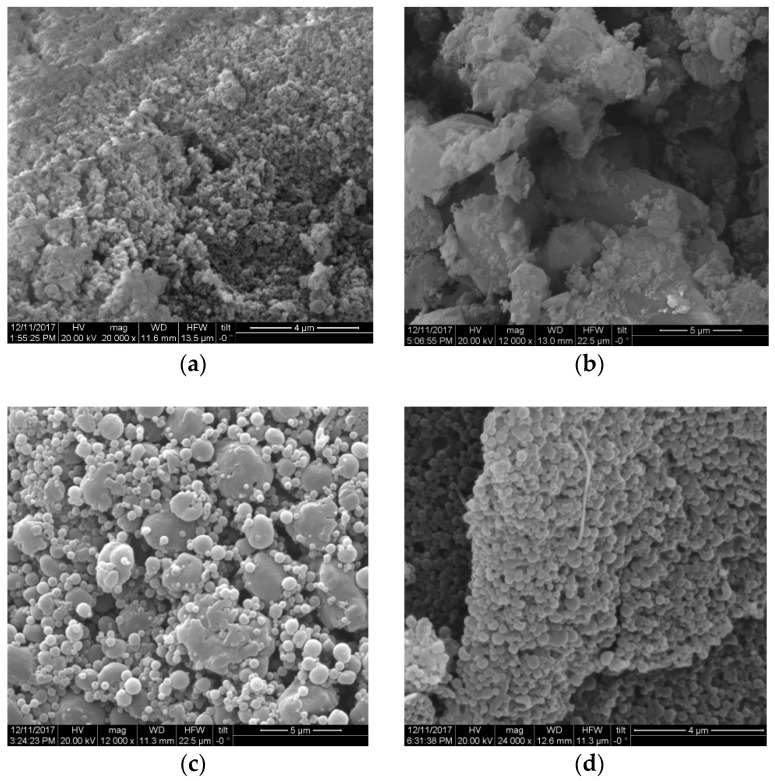
Morphology of lignin isolated with traditional OS pretreatment with 60% *v/v* ethanol content (**a**) and after homogenization at 500 bar in ethanol/water solution with ethanol content of (**b**) 0% *v/v*; (**c**) 50% *v/v*; and (**d**) 75% *v/v*.

An analogous positive effect of homogenization was observed with OS lignin (Figure 5a). There, homogenization facilitated the formation of defined spherical particles from initially unshaped and amorphous lignin. Similar to HOS-SE lignin, homogenization in the absence of ethanol (with 100% *v/v* water) failed to improve lignin morphology (Figure 5b). In contrast, homogenization with 50% *v/v* ethanol promoted the formation of spherical particles of various sizes (Figure 5c), which was further improved in terms of shape and size homogeneity when ethanol was increased to 75% *v/v* (Figure 5d). At these homogenization conditions, nanoparticle diameter varied between approximately 130 nm and 350 nm, with some of them being even smaller (Figure 5d). Comparable results were obtained by homogenization of OS lignin from the 50% *v/v* pretreatment.

The above observations were also confirmed by determining particle size with dynamic light scattering (DLS) (Table 1). Volume and intensity distributions of particle sizes of nanoparticles are shown in Supplementary Material, Figure S1. The results were in accordance with scanning electron microscopy (SEM) images and confirmed a reduction in particle size after homogenization. Increasing ethanol content during homogenization resulted in nanoparticles of lower diameter, in both OS and HOS-SE lignin samples (when spherical particles were formed). 

DLS served also to determine surface charge (zeta potential) of lignin samples forming spherical particles. Zeta potential is often used to predict colloidal stability [36]. High positive or negative zeta potential values indicate adequate electrical double layer repulsion between suspended nanoparticles, preventing their aggregation. The zeta potential is often measured on lignin nanoparticles [37], which generally exhibit negative values, a fact that can be attributed to the negative charge of phenols, as well as (partly) to the adsorption of hydroxyl ions [28,37,38,39]. In the present study, we observed a significant effect of ethanol content during pretreatment of HOS-SE on the zeta potential. Specifically, the surface charges on particles of HOS-SE lignin (prior to homogenization) isolated with 50% and 60% *v/v* ethanol were −30.4 ± 0.8 mV and −30.2 ± 1.8 mV, respectively. In contrast, at 70% *v/v* ethanol content, the zeta potential was less negative. This reduced zeta potential value could partially explain why, as noted by SEM, lignin particles isolated with HOS-SE using 50% *v/v* and 60% *v/v* had a well-formed spherical shape (Figure 2a,b), whereas lignin isolated with 70% *v/v* ethanol formed less defined spherical particles, which tended to create amorphous aggregates (Figure 2c).

In general, the homogenization post-treatment step resulted in the formation of more stable particles when compared to the initial lignin samples. Zeta potential values of the nanoparticle dispersion formed with HOS-SE and OS lignin at 60% *v/v* ethanol content after homogenization at 75% *v/v* ethanol were −39.3 ± 0.8 and −37.2 ± 1.9 mV, respectively (Table 1). These values indicate that such lignin particles were relatively stable in water [28]. This characteristic, combined with their well-distributed particle size and spherical shape, renders these lignin nanoparticles suitable for different applications, such as the production of nanocomposite films [38] or as drug carriers [40].

The same positive effect of homogenization on nanoparticle formation was observed in a previous study, whereby initially amorphous lignin was re-arranged and formed spherical nanoparticles [41]. However, it should be noted that optimal ethanol content for the formation of spherical nanoparticles was 50% *v/v* in that study. In general, the solvent:water ratio plays an important role in the formation of lignin nanoparticles, as these tend to form a hydrophobic core surrounded by a hydrophilic shell [42]. During self-assembly and formation of lignin nanoparticles, organic solvent content is gradually reduced by adding water, leading to hydrophobic aggregation of lignin molecules and subsequent sphere formation when water content surpasses a critical value [29,42]. A similar process for the formation of spherical lignin particles has also been proposed to occur during homogenization, whereby abundant hydrophilic moieties are constantly exposed to ethanol, resulting in the formation of spheres [25]. The optimal solvent:water ratio can therefore vary and is dependent on many factors, such as lignin chemical structure (the presence of hydrophilic moieties, their relative abundance, etc.), the type of solvent used, or the process operational parameters (speed of water content increase, pressure, etc.), all of which have an important effect on particle formation [28,29,42,43].

**Figure 6:**
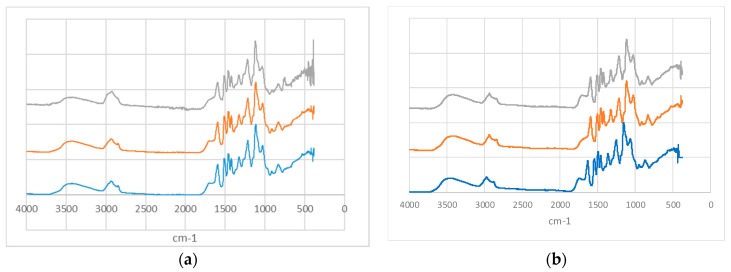
FT-IR spectra of (**a**) HOS-SE and (**b**) OS lignin isolated with 60% *v/v* ethanol, prior to homogenization (blue line), homogenized with 50% *v/v* ethanol (orange line) and 75% ethanol (grey line).

In the present study, we show that homogenization of lignin in the presence of ethanol and water solutions can promote the formation of micro- and nanoparticles. Importantly, we report that ethanol content affects their size and homogeneity. In this context, it is also essential that the lignin remains intact during the homogenization step and does not undergo chemical modifications or decomposition. To verify the integrity of lignin, we collected Fourier-transform infrared (FT-IR) spectra (Figure 6). These revealed various peaks characteristic of lignin, such as O–H stretching of aliphatic and phenolic OH, C–H stretching in aromatic methoxy, methyl, and methylene groups, C=O stretching, aromatic skeletal ring vibration, C–C aromatic skeletal ring vibration, C–H asymmetric deformation in methyl and methylene groups, and aromatic skeletal ring vibrations [43,44]. The spectra for lignin prior and after homogenization appeared similar, as indicated by the absence of changes in the number of peaks (disappearance or appearance of new peaks) or major peak shifts following homogenization. Based on this, we concluded that the homogenization did not have any major effect on lignin chemical structure. Analogous results, and consequent lack of notable changes, were observed in the FT-IR spectra of other homogenized HOS-SE and OS lignins (Supplementary Material, Figure S2).

## 3. Materials and Methods 

### 3.1. Materials

Birch chips (*Betula pendula* L.) from mills in Northern Sweden were used as feedstock for the present study. The chips were air-dried and milled in a knife mill (Retsch SM 300, Retsch GmbH, Haan, Germany) through a 1-mm screen. Milled chips were stored at room temperature until use.

### 3.2. Lignin Preparation

Lignin was isolated from birch chips by employing two fractionation processes, namely OS and HOS-SE. OS was performed in an autoclave apparatus as described before [18]. The conditions employed were 182 °C for 1 h at an ethanol content of 50% *v/v* or 60% *v/v*. HOS-SE fractionation took place as previously described at 200 °C [9]. The fractionation parameters for the HOS-SE were 15 min with 60% *v/v* ethanol, with and without the addition of 1% *w/w*_biomass_ H_2_SO_4_, and 30 min with 70% *v/v* ethanol or 50% *v/v* ethanol [9]. After fractionation, pretreated solids were separated from the pretreated liquor and lignin was isolated from the liquor as previously described [9,18].

### 3.3. Lignin Homogenization

Homogenization of lignin took place in an APV 1000 Rannie Mini-Lab pressure homogenizer (Albertslund, Denmark). For this purpose, 1 g of lignin was mixed with 100 mL of ethanol-water solution (ethanol content 0%, 50%, and 75% *v/v*) in a magnetic stirrer for 8 h at room temperature. Homogenization took place at 500 bar and the solution was recycled five times through the homogenizer. After homogenization, samples were diluted with cold water to attain an ethanol content below 10% *v/v*, which reduced the solubility of lignin in the solution. The solutions were then placed in 50-mL plastic tubes and centrifuged at 7000 rpm for 15 min, after which the supernatant was discarded. Finally, the lignin pellet was collected and dried in a freeze dryer.

### 3.4. Lignin Particles Characterization

Lignin particles were observed for their shape and size in a FEI Quanta scanning electron microscope (Thermo Fischer Scientific, Waltham, MA, USA). Imaging was carried out at an accelerating voltage of 20 kV, under high-vacuum conditions. All samples were coated with gold prior to imaging. An overall amount of 10 different images were taken for each sample in order to obtain a representative picture of it. Image analysis for the determination of particle size was performed with the ImageJ software. DLS and zeta potential measurements were performed on a Zetasizer Nano Series (Malvern Panalytical, Malvern, UK), with a multipurpose titrator. Specifically, a particle solution of 0.05 mg/mL was used for the analysis, with water chosen as dispersant. Each data point represents the average value of three measurements, with 11–15 runs for each measurement. FT-IR spectra were obtained on a Perkin Elmer Spectrum 100 Spectrometer (Waltham, MA, USA); the spectra were scanned over the range 4000–400 cm^−1^. The FTIR spectra presented are the average of 3 measurements.

## 4. Conclusions

The present study demonstrates that organosolv lignin can serve as a promising raw material for the preparation of lignin nanoparticles using a simple mechanical method. The technique used for biomass fractionation plays an important role in the formation of lignin particles. Accordingly, use of a newly established hybrid organosolv-steam explosion method allowed for the recovery of micro-particles directly after pretreatment. Operational parameters of organosolv fractionation affect the formation of lignin micro-particles at the end of the pretreatment. Crucially, homogenization can facilitate the formation of spherical lignin micro- and nanoparticles, even from initially amorphous lignin. The ethanol content during the homogenization step affects particle size, which tends to decrease as the ethanol content increases to 75% *v/v*. Lignin nanoparticles with an average size as low as 200 nm can be obtained under optimal conditions, demonstrating that tuning of the operational parameters for the particle preparation is a crucial step to get high quality lignin nanoparticles. Finally, the particles’ zeta potential charge indicates that they have good colloidal stability, and FT-IR spectra reveal that lignin molecular integrity is unaffected by the homogenization step.

## Figures and Tables

**Table 1:** Determination of particle size, polydispersity index, and zeta potential of lignin micro- and nanoparticles isolated in the present study.

Lignin Sample	Size (nm)	PDI	Zeta Potential (mV)
HOS-SE, 50% *v/v* EtOH, w/o homogenization	3101 ± 81	0.635 ± 0.084	−30.4 ± 0.8
HOS-SE, 50% *v/v* EtOH, homogenization with 0% *v/v* EtOH	2002 ± 52	0.248 ± 0.016	−47.1 ± 0.6
HOS-SE, 50% *v/v* EtOH, homogenization with 50% *v/v* EtOH	650 ± 9	0.164 ± 0.027	−37.1 ± 1.2
HOS-SE, 50% *v/v* EtOH, homogenization with 75% *v/v* EtOH	488 ± 14	0.486 ± 0.011	−24.5 ± 0.6
HOS-SE, 60% *v/v* EtOH, w/o homogenization	4505 ± 326	0.285 ± 0.043	−30.2 ± 1.8
HOS-SE, 60% *v/v* EtOH, homogenization with 50% *v/v* EtOH	1165 ± 42	0.338 ± 0.029	−30.9 ± 0.5
HOS-SE, 60% *v/v* EtOH, homogenization with 75% *v/v* EtOH	825 ± 25	0.356 ± 0.002	−37.2 ± 1.9
HOS-SE, 70% *v/v* EtOH, w/o homogenization	2615 ± 999	1.000 ± 0.000	−19.6 ± 1.6
HOS-SE, 70% *v/v* EtOH, homogenization with 75% *v/v* EtOH	200 ± 4	0.341 ± 0.001	−30.8 ± 1.6
OS, 50% *v/v* EtOH, homogenization with 50% *v/v* EtOH	956 ± 10	0.413 ± 0.035	−38.0 ± 1.0
OS, 50% *v/v* EtOH, homogenization with 75% *v/v* EtOH	530 ± 972	0.502 ± 0.094	−35.4 ± 0.7
OS, 60% *v/v* EtOH, homogenization with 50% *v/v* EtOH	906 ± 125	0.548 ± 0.069	−20.5 ± 1.1
OS, 60% *v/v* EtOH, homogenization with 75% *v/v* EtOH	834 ± 27	0.457 ± 0.072	−39.3 ± 0.8

Analysis with DLS was performed only on lignin samples that formed spherical particles.

## Supplementary Materials

The following are available online, Figure S1: Intensity (I) and volume (V) distributions of particle sizes of nanoparticles, Figure S2: FT-IR graphs of lignin nanoparticles.

## References

[1] Katsimpouras C., Zacharopoulou M., Matsakas L., Rova U., Christakopoulos P., Topakas E. (2017). Sequential high gravity ethanol fermentation and anaerobic digestion of steam explosion and organosolv pretreated corn stover. Bioresour. Technol..

[2] Wan C., Zhou Y., Li Y. (2011). Liquid hot water and alkaline pretreatment of soybean straw for improving cellulose digestibility. Bioresour. Technol..

[3] Sahoo D., Ummalyma S.B., Okram A.K., Pandey A., Sankar M., Sukumaran R.K. (2018). Effect of dilute acid pretreatment of wild rice grass (*Zizania latifolia*) from Loktak Lake for enzymatic hydrolysis. Bioresour. Technol..

[4] Matsakas L., Christakopoulos P. (2013). Fermentation of liquefacted hydrothermally pretreated sweet sorghum bagasse to ethanol at high-solids content. Bioresour. Technol..

[5] Nitsos C., Matsakas L., Triantafyllidis K., Rova U., Christakopoulos P. (2017). Investigation of different pretreatment methods of Mediterranean-type ecosystem agricultural residues: Characterisation of pretreatment products, high-solids enzymatic hydrolysis and bioethanol production. Biofuels.

[6] Ragauskas A.J., Beckham G.T., Biddy M.J., Chandra R., Chen F., Davis M.F., Davison B.H., Dixon R.A., Gilna P., Keller M. (2014). Lignin valorization: Improving lignin processing in the biorefinery. Science.

[7] Becker J., Boles E. (2003). A modified Saccharomyces cerevisiae strain that consumes L-arabinose and produces ethanol. Appl. Environ. Microbiol..

[8] Öhgren K., Bengtsson O., Gorwa-Grauslund M.F., Galbe M., Hahn-Hägerdal B., Zacchi G. (2006). Simultaneous saccharification and co-fermentation of glucose and xylose in steam-pretreated corn stover at high fiber content with Saccharomyces cerevisiae TMB3400. J. Biotechnol..

[9] Matsakas L., Nitsos C., Raghavendran V., Yakimenko O., Persson G., Olsson E., Rova U., Olsson L., Christakopoulos P. (2018). A novel hybrid organosolv: Steam explosion method for the efficient fractionation and pretreatment of birch biomass. Biotechnol. Biofuels.

[10] Liu W.-J., Jiang H., Yu H.-Q. (2015). Thermochemical conversion of lignin to functional materials: A review and future directions. Green Chem..

[11] Beckham G.T., Johnson C.W., Karp E.M., Salvachúa D., Vardon D.R. (2016). Opportunities and challenges in biological lignin valorization. Curr. Opin. Biotechnol..

[12] Azadi P., Inderwildi O.R., Farnood R., King D.A. (2013). Liquid fuels, hydrogen and chemicals from lignin: A critical review. Renew. Sustain. Energy Rev..

[13] Schutyser W., Renders T., Van den Bosch S., Koelewijn S.-F., Beckham G.T., Sels B.F. (2018). Chemicals from lignin: An interplay of lignocellulose fractionation, depolymerisation, and upgrading. Chem. Soc. Rev..

[14] Duval A., Lawoko M. (2014). A review on lignin-based polymeric, micro- and nano-structured materials. React. Funct. Polym..

[15] Sun Z., Fridrich B., De Santi A., Elangovan S., Barta K. (2018). Bright Side of Lignin Depolymerization: Toward New Platform Chemicals. Chem. Rev..

[16] Smit A., Huijgen W. (2017). Effective fractionation of lignocellulose in herbaceous biomass and hardwood using a mild acetone organosolv process. Green Chem..

[17] Wildschut J., Smit A.T., Reith J.H., Huijgen W.J.J. (2013). Ethanol-based organosolv fractionation of wheat straw for the production of lignin and enzymatically digestible cellulose. Bioresour. Technol..

[18] Nitsos C., Stoklosa R., Karnaouri A., Vörös D., Lange H., Hodge D., Crestini C., Rova U., Christakopoulos P. (2016). Isolation and characterization of organosolv and alkaline lignins from hardwood and softwood biomass. ACS Sustain. Chem. Eng..

[19] Matsakas L., Nitsos C., Vörös D., Rova U., Christakopoulos P. (2017). High-titer methane from organosolv-pretreated spruce and birch. Energies.

[20] Alvira P., Tomás-Pejó E., Ballesteros M., Negro M.J. (2010). Pretreatment technologies for an efficient bioethanol production process based on enzymatic hydrolysis: A review. Bioresour. Technol..

[21] Zhao X., Cheng K., Liu D. (2009). Organosolv pretreatment of lignocellulosic biomass for enzymatic hydrolysis. Appl. Microbiol. Biotechnol..

[22] Rinaldi R., Jastrzebski R., Clough M.T., Ralph J., Kennema M., Bruijnincx P.C.A., Weckhuysen B.M. (2016). Paving the way for lignin valorisation: Recent advances in bioengineering, biorefining and catalysis. Angew. Chem. Int. Ed..

[23] Beisl S., Miltner A., Beisl S., Miltner A., Friedl A. (2017). Lignin from Micro-to Nanosize: Production methods. Int. J. Mol. Sci..

[24] Beisl S., Loidolt P., Miltner A., Harasek M., Friedl A. (2018). Production of micro- and nanoscale lignin from wheat straw using different precipitation setups. Molecules.

[25] Rao X., Liu Y., Zhang Q., Chen W., Liu Y., Yu H. (2017). Assembly of Organosolv Lignin Residues into Submicron Spheres: The Effects of Granulating in Ethanol/Water Mixtures and Homogenization. ACS Omega.

[26] Nair S.S., Sharma S., Pu Y., Sun Q., Pan S., Zhu J.Y., Deng Y., Ragauskas A.J. (2014). High shear homogenization of lignin to nanolignin and thermal stability of nanolignin-polyvinyl alcohol blends. Chem. Sustain. Chem..

[27] Garcia Gonzalez M.N., Levi M., Turri S., Griffini G. (2017). Lignin nanoparticles by ultrasonication and their incorporation in waterborne polymer nanocomposites. J. Appl. Polym. Sci..

[28] Lievonen M., Valle-Delgado J.J., Mattinen M.-L., Hult E.-L., Lintinen K., Kostiainen M.A., Paananen A., Szilvay G.R., Setälä H., Österberg M. (2016). A simple process for lignin nanoparticle preparation. Green Chem..

[29] Li H., Deng Y., Liu B., Ren Y., Liang J., Qian Y., Qiu X., Li C., Zheng D. (2016). Preparation of nanocapsules via the self-assembly of kraft lignin: A totally green process with renewable resources. ACS Sustain. Chem. Eng..

[30] Chum H.L., Johnson D.K., Black S.K. (1990). Organosolv Pretreatment for enzymatic hydrolysis of poplars. 2. catalyst effects and the combined severity parameter. Ind. Eng. Chem. Res..

[31] Huijgen W.J.J., Smit A.T., Reith J.H., Uil H. (2011). den Catalytic organosolv fractionation of willow wood and wheat straw as pretreatment for enzymatic cellulose hydrolysis. J. Chem. Technol. Biotechnol..

[32] Sturgeon M.R., Kim S., Lawrence K., Paton R.S., Chmely S.C., Nimlos M., Foust T.D., Beckham G.T. (2014). A Mechanistic investigation of acid-catalyzed cleavage of aryl-ether linkages: Implications for lignin depolymerization in acidic environments. ACS Sustain. Chem. Eng..

[33] Kobayashi T., Kohn B., Holmes L., Faulkner R., Davis M., MacIel G.E. (2011). Molecular-level consequences of biomass pretreatment by dilute sulfuric acid at various temperatures. Energy Fuels.

[34] Richter A.P., Bharti B., Armstrong H.B., Brown J.S., Plemmons D., Paunov V.N., Stoyanov S.D., Velev O.D. (2016). Synthesis and characterization of biodegradable lignin nanoparticles with tunable surface properties. Langmuir.

[35] Li H., Deng Y., Liang J., Dai Y., Liu B., Ren Y., Qiu X., Li C. (2016). Direct preparation of hollow nanospheres with kraft lignin: A facile strategy for effective utilization of biomass waste. BioResources.

[36] Xu R., Wu C., Xu H. (2007). Particle size and zeta potential of carbon black in liquid media. Carbon.

[37] Frangville C., Rutkevičius M., Richter A.P., Velev O.D., Stoyanov S.D., Paunov V.N. (2012). Fabrication of environmentally biodegradable lignin nanoparticles. Chem. Phys. Chem..

[38] Tian D., Hu J., Bao J., Chandra R.P., Saddler J.N., Lu C. (2017). Lignin valorization: Lignin nanoparticles as high-value bio-additive for multifunctional nanocomposites. Biotechnol. Biofuels.

[39] Wei Z., Yang Y., Yang R., Wang C. (2012). Alkaline lignin extracted from furfural residues for pH-responsive Pickering emulsions and their recyclable polymerization. Green Chem..

[40] Sipponen M.H., Lange H., Ago M., Crestini C. (2018). Understanding lignin aggregation processes. A case study: Budesonide entrapment and stimuli controlled release from lignin nanoparticles. ACS Sustain. Chem. Eng..

[41] Maniet G., Schmetz Q., Jacquet N., Temmerman M., Gofflot S., Richel A. (2017). Effect of steam explosion treatment on chemical composition and characteristic of organosolv fescue lignin. Ind. Crops Prod..

[42] Qian Y., Deng Y., Qiu X., Li H., Yang D. (2014). Formation of uniform colloidal spheres from lignin, a renewable resource recovered from pulping spent liquor. Green Chem..

[43] Xiong F., Han Y., Wang S., Li G., Qin T., Chen Y., Chu F. (2017). Preparation and formation mechanism of size-controlled lignin nanospheres by self-assembly. Ind. Crops Prod..

[44] Boeriu C.G., Bravo D., Gosselink R.J.A., Van Dam J.E.G. (2004). Characterisation of structure-dependent functional properties of lignin with infrared spectroscopy. Ind. Crops Prod..

