# Supplementary material for: Formation of Lignin Nanoparticles by Combining Organosolv Pretreatment of Birch Biomass and Homogenization Processes
Source: Molecules. 2018 Jul 23;23(7):1822. doi: 10.3390/molecules23071822 (PMC6100471; doi:10.3390/molecules23071822)

Article

Formation of Lignin Nanoparticles by Combining Organosolv Pretreatment of Birch Biomass and Homogenization Processes

Leonidas Matsakas [1], Anthi Karnaouri [1], Andrzej Cwirzen [2], Ulrika Rova [1] and Paul Christakopoulos [1,*]

[1] Biochemical Process Engineering, Division of Chemical Engineering, Department of Civil, Environmental and Natural Resources Engineering, Luleå University of Technology, 971-87 Luleå, Sweden; leonidas.matsakas@ltu.se (L.M) anthi.karnaouri@ltu.se (A.K.); ulrika.rova@ltu.se (U.R.)

[2] Structural Engineering, Division of Structural and Fire Engineering, Department of Civil, Environmental and Natural Resources Engineering, Luleå University of Technology, 971-87 Luleå, Sweden. andrzej.cwirzen@ltu.se (A.C.)

* Correspondence: paul.christakopoulos@ltu.se; Tel.: +46-9204-92510

Supplementary Material

Figure S1. Volume (*V*) and intensity (I) distributions of particle sizes of nanoparticles.

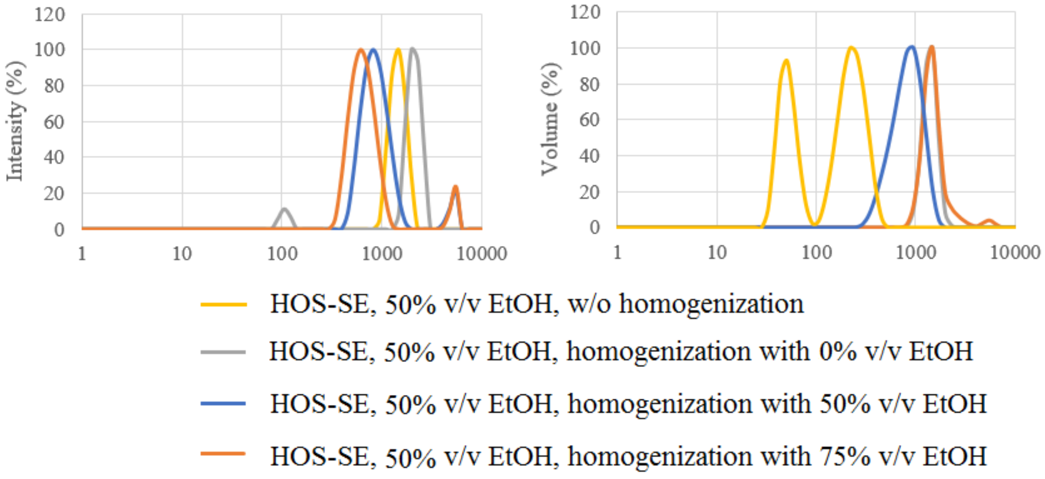

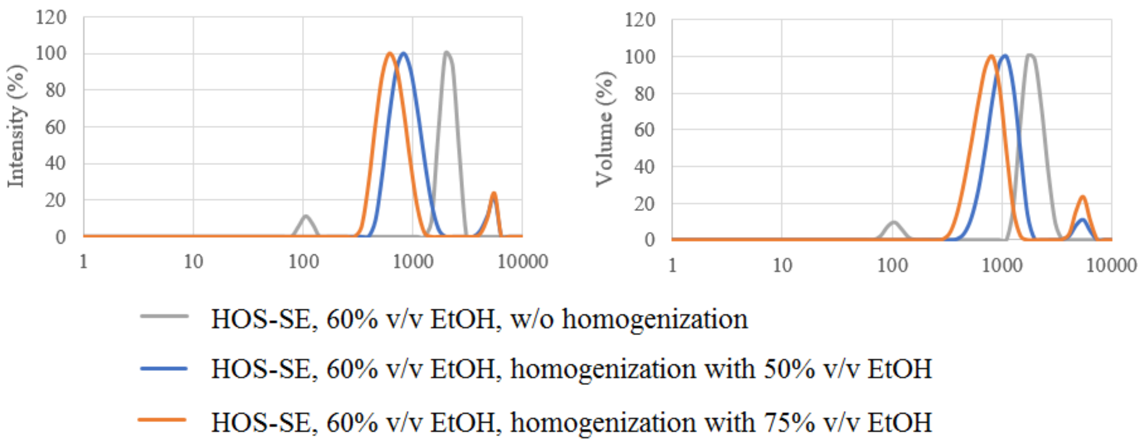

Intensity (%)
Volume (%)
120
100
80
60
40
20
0
1
10
100
1000
10000
HOS-SE, 60% v/v EtOH, w/o homogenization
HOS-SE, 60% v/v EtOH, homogenization with 50% v/v EtOH
HOS-SE, 60% v/v EtOH, homogenization with 75% v/v EtOH

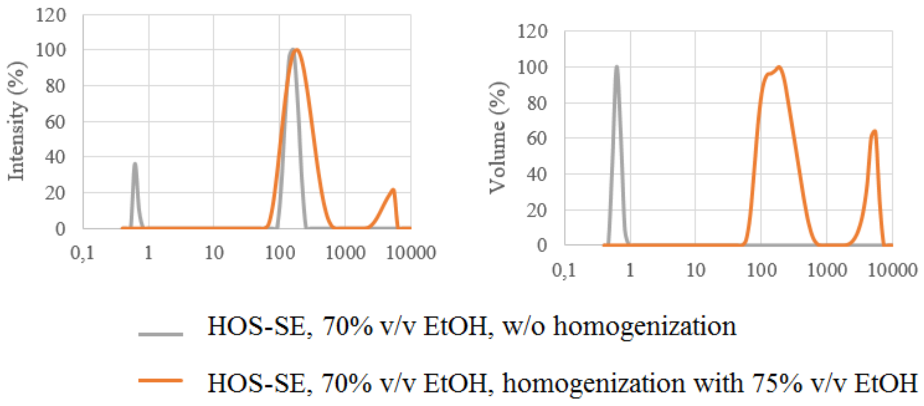

Intensity (%)
Volume (%)
120
100
80
60
40
20
0
0,1
1
10
100
1000
10000
HOS-SE, 70% v/v EtOH, w/o homogenization
HOS-SE, 70% v/v EtOH, homogenization with 75% v/v EtOH

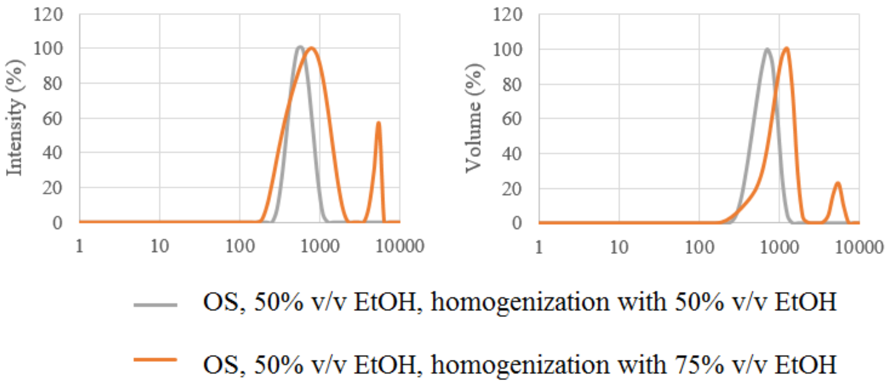

Intensity (%)
Volume (%)
120
100
80
60
40
20
0
1
10
100
1000
10000
OS, 50% v/v EtOH, homogenization with 50% v/v EtOH
OS, 50% v/v EtOH, homogenization with 75% v/v EtOH

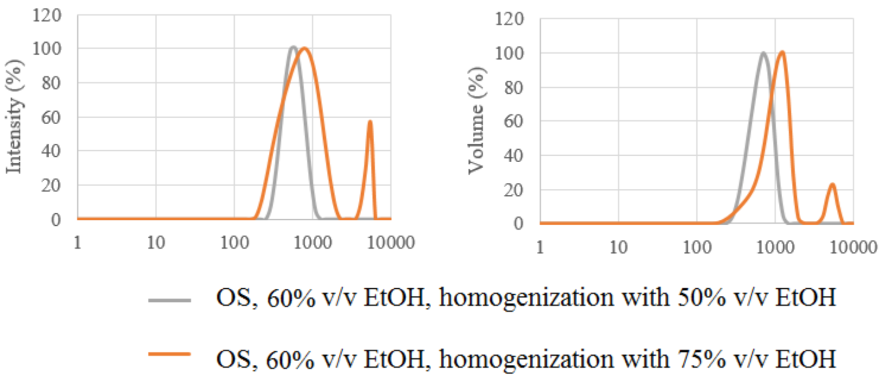

Figure S2. FT-IR graphs of lignin nanoparticles.

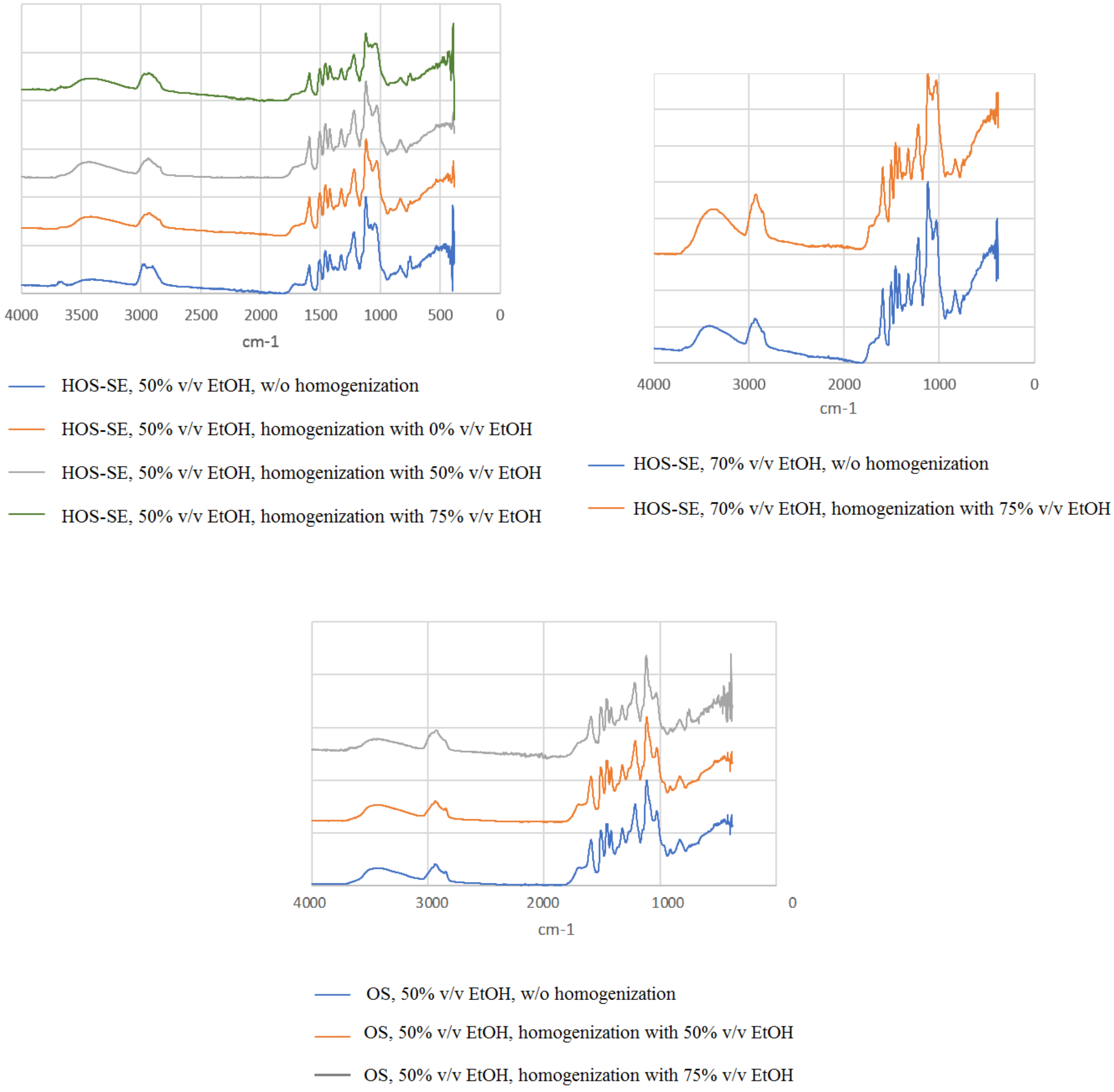

Supplement: Supplementary file 1 [file molecules-23-01822-s001.pdf]
